# ZMIZ1 Preferably Enhances the Transcriptional Activity of Androgen Receptor with Short Polyglutamine Tract

**DOI:** 10.1371/journal.pone.0025040

**Published:** 2011-09-20

**Authors:** Xiaomeng Li, Chunfang Zhu, William H. Tu, Nanyang Yang, Hui Qin, Zijie Sun

**Affiliations:** 1 Department of Urology, Stanford University School of Medicine, Stanford, California, United States of America; 2 Department of Genetics, Stanford University School of Medicine, Stanford, California, United States of America; 3 The Key Laboratory of Molecular Epigenetics of MOE, Institute of Genetics and Cytology, Northeast Normal University, Changchun, The People's Republic of China; Clermont Université, France

## Abstract

The androgen receptor (AR) is a ligand-induced transcription factor and contains the polyglutamine (polyQ) tracts within its N-terminal transactivation domain. The length of polyQ tracts has been suggested to alter AR transcriptional activity in prostate cancer along with other endocrine and neurologic disorders. Here, we assessed the role of ZMIZ1, an AR co-activator, in regulating the activity of the AR with different lengths of polyQ tracts as ARQ9, ARQ24, and ARQ35 in prostate cancer cells. ZMIZ1, but not ZMIZ2 or ARA70, preferably augments ARQ9 induced androgen-dependent transcription on three different androgen-inducible promoter/reporter vectors. A strong protein-protein interaction between ZMIZ1 and ARQ9 proteins was shown by immunoprecipitation assays. In the presence of ZMIZ1, the N and C-terminal interaction of the ARQ9 was more pronounced than ARQ24 and ARQ35. Both Brg1 and BAF57, the components of SWI/SNF complexes, were shown to be involved in the enhancement of ZMIZ1 on AR activity. Using the chromatin immunoprecipitation assays (ChIP), we further demonstrated a strong recruitment of ZMIZ1 by ARQ9 on the promoter of the *prostate specific antigen* (*PSA*) gene. These results demonstrate a novel regulatory role of ZMIZ1 in modulating the polyQ tract length of AR in prostate cancer cells.

## Introduction

Androgen signaling is mainly mediated through the androgen receptor (AR) and plays a critical role in male sexual development and in normal and malignant prostatic cell growth and survival [1]. AR is a member of the steroid hormone receptor superfamily. Like other steroid hormone receptors, AR contains four functional domains: an N-terminal transactivation domain (NTD), a central DNA-binding domain (DBD), a hinge region between the DBD and LBD domains, and a C-terminal ligand-binding domain (LBD) [2]. The NTD of human AR is encoded by exon 1, containing polyglutamine (polyQ) tract. It has been suggested that this unique sequence structure modulates the activity of AR mediated transcription. The biological significance of the polyQ tract abnormality has been explored in human diseases. Expansion of the polyQ length within the AR NTD directly links to spinal and bulbar muscular atrophy/Kennedy's disease (SBMA), a rare, X-linked, adult onset, neurodegenerative disorder [3], [4], [5]. Male patients with this disorder often have symptoms of partial androgen insensitivity indicative of aberrant AR function, such as gynecomastia and testicular atrophy [6], [7]. The length of the polyQ repeats within AR has also been implicated in other human disorders, including the inherited form of androgen insensitivity (AIS), hereditary hearing impairment, schizophrenia, and benign prostatic hyperplasia [7], [8], [9]. Conversely, multiple lines of evidence have shown that the length of the AR polyQ tract is inversely correlated with the risk of developing prostate cancer, age of onset, and risk of advanced disease at diagnosis [10], [11], [12].

The AR mediated transcription is modulated in a ligand-dependent manner and facilitated through multiple co-regulators [13], [14]. Similar to other receptors, the unbound AR forms a complex with heat-shock proteins, HSPs [15]. Upon binding to ligands, the AR dissociates from the HSPs and translocates into the nucleus, where it binds to the androgen response element (ARE), recruits other transcriptional cofactors, and induces ligand-dependent transcription [2]. The recruitment of transcriptional co-regulators and the basal transcriptional machinery is essential for conferring the full transcriptional activity of AR. An inverse correlation between the length of the polyQ region and the activity of AR-mediated transcription has been implicated [16]. It has been shown that increasing polyQ length negatively affects AR co-activators, p160, mediated co-activation of the AR [17], [18], [19]. AR proteins containing a shortened polyQ length showed stronger interaction with p160 co-activators and components of the SWI/SNF chromatin-remodeling complexes than the wild type AR [19]. Ras-related nuclear protein/ARA24 was identified as an AR co-activator that interacts with the AR polyQ region [20]. The activity of ARA24 was reduced or diminished with the polyQ expansion within AR. These data suggest that the interactions between AR co-regulators and the polyQ region of AR affect AR-mediated transcription.

ZMIZ1, original named Zimp10 (zinc finger-containing, Miz1, PIAS-like protein on chromosome 10), has been demonstrated as an AR co-activator [21]. A protein-protein interaction was identified between the AR NTD and the central region of ZMIZ1. Sequence analysis revealed that ZMIZ1shares a highly conserved SP-RING/Miz domain with members of the PIAS family [22]. In addition to the SP-RING/Miz domain, ZMIZ1 also contains a nuclear localization sequence (NLS) and two proline-rich regions [21]. Importantly, a strong intrinsic transactivation domain was identified within the C-terminal region of ZMIZ1, through which ZMIZ1 augments AR-mediated transcription [21]. ZMIZ1 has also been shown to co-localize with AR and small ubiquitin-like modifier SUMO-1 and forms a protein complex at replication foci in the nucleus [21], [23]. To further understand the role of ZMIZ1 in regulating AR-mediated transcription, we investigated the role of ZMIZ1 in modulating the transcriptional activity of AR containing different lengths of polyQ tract. Intriguingly, ZMIZ1 showed stronger enhancement of ligand-induced transcription with the AR proteins possessing the short polyQ tracts. Using different biochemical and functional approaches, we further assessed the effect of ZMIZ1 in the interaction between the N- and C-terminuses of AR and the potential mechanisms underlying ZMIZ1 in modulating AR-mediated transcription.

## Materials and Methods

### DNA plasmids

The human AR plasmid, pSV-hAR, was kindly provided by Dr Albert Brinkmann (Erasmus University, Rotterdam, The Netherlands). The AR plasmids containing different polyQ tracts were generously supplied by Dr. Gerhard A. Coetzee (USC, Los Angles, California). The DNA fragments of the AR NTD with different polyQ tracts, LBD, and DBD were generated by PCR approaches with appropriate primers and then subcloned in-frame into either the pGEX4T1 vector for producing GST fusion proteins, or pcDNA3.1, pSV, and pLentiSuper expression vectors [24]. The human Brg1 and BAF57 expression vectors were gifts from Dr. Gerald Crabtree (Stanford University) and the expression vector for ARA70 and the pARE-luc reporter were kindly provided by Dr. Chawnshang Chang [25]. pcDNA3-FLAG-ZMIZ1, pcDNA3-FLAG-ZMIZ2, and pcDNA3-FLAG-ARA70 were made in the lab as described previously [21], [26]. The pPSA7kb-luc was kindly provided by Dr. Jan Trapman [27]. MMTV-luc was kindly provided Dr. Richard Pestell [28]. pSV-β-galactosidase (β-gal) reporter plasmid (Promega, Madison, WI), was used in this study as an internal control.

### Cell cultures, transfections, lentivirus production, and reporter assays

DU145 and PC3, human prostate cancer cell lines from the American Tissue Culture Collection (ATCC, HTB-81 and CRL-1435), CV-1, a monkey kidney cell line [29], and SW13, a human adrenal carcinoma cell line [30], were maintained in Dulbecco's modified Eagle's medium (DMEM) supplemented with 5 or 10% fetal bovine serum (HyClone, Denver, CO). Lentiviruses were generated by cotransfection of pLenti-puro-ARQ9, pLenti-puro-ARQ24, or pLenti-puro-ARQ35, with pCMV-dR8.91 and pMD2.G-VSVG into HKE293 cells at a ratio of 3∶2∶1 using a LipofectAMINE2000 kit (Invitrogen) as described previously [31], [32]. The media were replaced at 6 hr post transfection and then collected 36–40 hr later. The viral supernatant was centrifuged briefly to remove cellular debris and stored at −80°C. Lentivirus infection was carried out in the presence of 6 µg/ml polybrene, and then selected with puromycin (Sigma) after 48 hrs. DU145 cells with stable expression of different AR polyQ length proteins were selected and tested for the expression of AR proteins.

Transient transfections were carried out using a Lipofectamine 2000 transfection kit (Invitrogen, Carlsbad, CA). Approximately 1.5–2×10^4^ cells were plated in a 48-well plate 16 hr before transfection. Approximately 200 ng of total plasmid DNA was used per well. The total amount of plasmid per well was kept constant by adding empty pcDNA3 or pBluescript vectors (Stratagene, CA) as needed. Individual transfection experiments were repeated a minimum of three times in triplicate with at least two different preparations of plasmid DNA. Approximately 12–16 hr after transfection, the cells were fed medium containing 5% charcoal-stripped fetal calf serum (FCS, HyClone, Denver, CO) in the presence or absence of dihydrotestosterone (DHT). Whole cell lysates were prepared and used for luciferase and β-gal assays as described previously [33]. Luciferase activity is measured after a 5 sec delay following injection of 50 µl luciferase buffer and 50 µl luciferin into 50 µl cell lysate by the dual injector luminometer, according to manufacturer's instruction (Analytical Luminesence Lab., San Diego, CA). The relative luciferase unit (RLU) from individual transfections was normalized by measurement of β-galactosidase (luciferase/β-galactosidase) activity expressed from a cotransfected plasmid in the same samples. RLUs were determined from three independent transfection experimetns and are presented as the mean ± SEM of the triplicate transfections.

### Immunoprecipitation and Western Blotting

DU145 cells infected with pLenti-puro-ARQ9, ARQ24, or ARQ35 were subsequently transfected with the FLAG-tagged hZMIZ1 expression vectors. Transfected cells were cultured in the presence of 10 nM DHT for 48 h and then harvested in a buffer containing 0.5% Nonidet P-40, 150 mM NaCl, 2 mM MgCl2, 50 mM HEPES-KOH (pH 7.4), 1 mM EDTA, 5% glycerol, 1 mM dithiothreitol, 0.5 mM phenylmethylsulfonyl fluoride, 25 mM NaF. Lysates were clarified by incubation on ice and centrifugation for 5 min. Four hundred µl of clarified lysate from each sample was precleared for 20 min with 10 µl of protein-A sepharose beads bound to 1 µg of normal mouse IgG (Pharmacia). Precleared lysates were then incubated with pre-equilibrated protein-A-Sepharose beads with either normal rabbit IgG or AR polyclonal antibody (Cat#: 06-680, Upstate Biotechnology, NY) at 4°C for 3 h. The beads were collected by centrifugation, and gently washed 3 times with the same buffer as described above. Proteins were eluted by boiling in SDS-sample buffer and resolved on a 10% SDS-PAGE, and transferred onto a nitrocellulose membrane. Membranes were probed with AR (sc-816, Santa Cruz Biotechnology) or FLAG (F3165, Sigma) antibody at 1∶500 or 1∶1000 dilutions, respectively. Anti-rabbit or mouse IgG conjugated to horseradish peroxidase was used as secondary antibodies at the dilution 1∶1000 (Cat#170-6515 or Cat#170-6516, Bio-Rad, Hercules, CA). Detection was performed with ECL reagents according to the manufacturer's protocol using ECL Hyperfilm (Amersham Biosciences).

### GST Pull down Assay

Glutathione *S*-transferase (GST) fusion proteins with the AR NTD domain of polyQ-ARQ9, Q24, Q35, the full length of ZMIZ1, and ARA70 were constructed in the pGEX-2TK vector (Amersham, Arlington Heights, IL). Expression and purification of GST fusion proteins were performed as described previously [33]. Equal amounts of GST fusion proteins coupled to glutathione-Sepharose beads were incubated with the radio-labeled proteins or whole cell lysates at 4°C for 4 h in a modified binding buffer (20 mM Tris-HCl, pH 7.8, 180 mM KCl, 0.5 mM EDTA, 5 mM MgCl2, 50 µM ZnCl2, 10% glycerol, 0.1% Nonidet P-40, 0.05% dry nonfat milk, 1 mM dithiothreitol, 0.5 mM phenylmethylsulfonyl fluoride) in the presence of 10 nM DHT. Beads were carefully washed 3 times with 500 µl of binding buffer and then analyzed by SDS-PAGE followed by autoradiography or immunoblotting with appropriate antibodies.

### Chromatin immunoprecipitation (ChIP) Assays

DU145 cells stably transfected with 7 Kb-PSA promoter plasmids were infected with different AR polyQ lentiviral vectors, and then subsequently transfected with FLAG-tagged hZMIZ1 expression vectors. Cells were selected with puromycin and G418 for at least 7 day. For ChIP assays, cells were replated and cultured in the medium containing 5% charcoal-stripped FCS in the presence or absence of 10 nM DHT at 37°C for 12 hours, and then washed with PBS. After the last wash with PBS, cells were treated with formaldehyde [34]. Cells were then collected and washed sequentially with cold PBS, Wash Buffer I (0.25% Triton X100, 10 mM EDTA, 0.5 mM EGTA and 10 mM HEPES, pH 6.5), and Wash Buffer II (200 mM NaCl, 1 mM EDTA, 0.5 mM EGTA and 10 mM HEPES, pH 6.5). Cells were lysed in buffer (1% SDS, 10 mM EDTA, 50 mM Tris pH 8.1, and protease inhibitors). The chromatin was sheared to an average size of 800 bp by sonication and diluted ten-fold in ChIP dilution buffer (2 mM EDTA, 150 mM NaCl, 20 mM Tris-HCl, pH 8.1, 1% Triton X-100), and then subjected to immunoprecipitation with an anti-AR antibody (Cat# 06-680, Upstate), anti-FLAG antibody (Cat#F3165, Sigma), or normal IgG for overnight at 4°C and recovered with Protein A Sepharose (Amersham). The immuno-complexes were eluted from the beads through incubation with 10× bead volume of elution buffer (1% SDS, 0.1 M NaHCO3). Crosslinks were reversed by incubating elution samples at 65°C for 6 hrs and chromatin fragments were purified with the PCR Purification Kit (Qiagen). ChIP and input DNA were analyzed by PCR using PSA promoter-specific primers reported previously [35]. AREI-forward: TCTGCCTTTGTCCCCTAGAT, reverse: AACCTTCATTCCCCAGGACT; AREII forward: AGGGATCAGGGAGTCTCACA, reverse: GCTAGCACTTGCTGTTCTGC; AREIII forward: CTGCTCAGCCTTTGTCTCTG, reverse: CAGATCCAGGCTTGCTTAC. Samples were also amplified with E/F primers for controls: forward: CTGTGCTTGGAGTTTACCTGA, and reverse: GCAGAGGTTGCAGTGAGCC.

## Results

### ZMIZ1 enhances the transcriptional activity of AR with shorter polyQ tract

**Figure 1:**
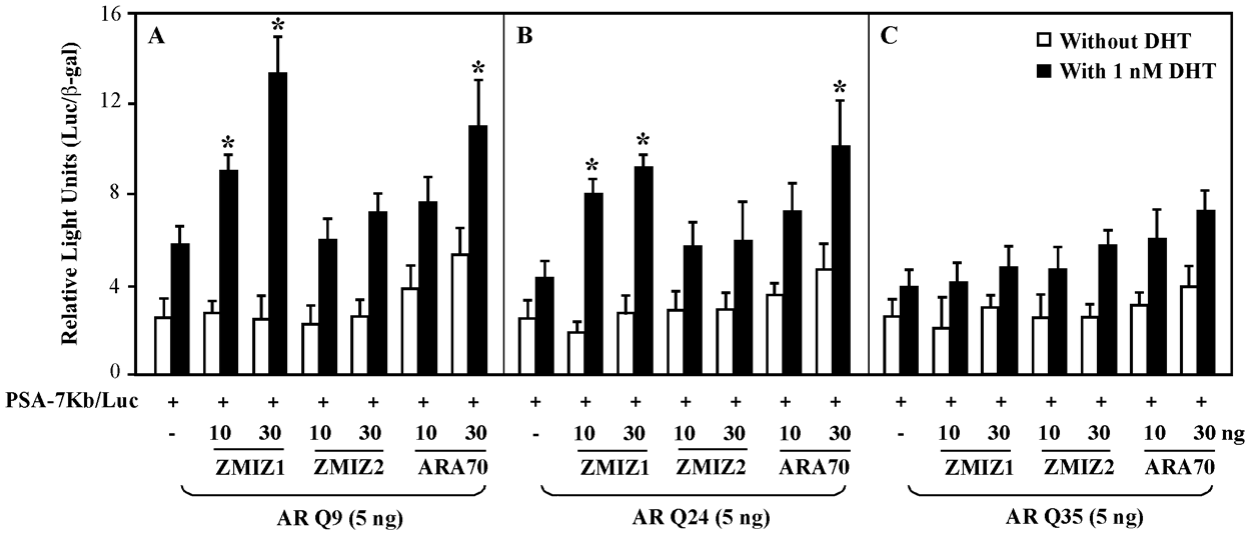
ZMIZ1 selectively enhances the shorter polyQ-AR mediated transcription. DU145 cells were transiently transfected in 48-well plates with 100 ng of 7 kb PSA-Luc, 25 ng of pSV40-β-gal, 5 ng of ARQ9, Q24, or Q35, and 10 or 30 ng of pcDNA3-FLAG-hZMIZ1, pcDNA3-FLAG-hZMIZ2, or pcDNA3-FLAG-ARA70 as where are indicated. The total amount of plasmids per well was normalized in all transfections by the addition of pcDNA3 empty vector. Twenty-four hours after transfection, cells were incubated with or without 1 nM DHT for another 24 hours and then whole cell lysates were prepared for assessment of luciferase and β-gal activities. Transfection experiments were repeated a minimum of three times in triplicate. Relative luciferase units (RLUs) were determined from three independent transfections and are presented as the mean ± SEM of the triplicate transfections. “*” indicates a statistically significant difference (p<0.05) between the samples co-expressed AR and other AR co-factors in the presence of 1 nM DHT.

Full activity of the ligand-induced AR mediated transcription is facilitated through the interactions between the AR and its co-regulators [13], [14]. Previous studies have shown that the length of AR polyQ repeats influences the transcriptional activity of AR [19], [36]. ZMIZ1 is an AR co-activator and interacts with the NTD of AR. In this study, we assessed the possible effect of ZMIZ1 on the transactivity of AR with different polyQ tracts. Expression vectors for the human AR cDNA containing 9, 24, and 35 polyQ tracts and for AR cofactors, including ZMIZ1, ZMIZ2, and ARA70, were co-transfected with an androgen-induced luciferase reporter driven by a 7 Kb PSA promoter-enhancer fragment into prostate cancer cell line, DU145. A ligand-induced transactivation on the PSA luciferase reporter was observed in all of the samples transfected with different AR polyQ length expression vectors whereas the samples transfected with ARQ9 showed the highest induction (Fig. 1). There is no significant change in expression of the above exogenous proteins in each sample (please see Fig. S1). Co-expression of ZMIZ1 with different AR polyQ vectors further increased the ligand-induced activity. Particularly, ZMIZ1 showed a significant enhancement on ARQ9 and Q24 mediated transcription in a dosage dependent manner but not on ARQ35 (Fig. 1A, 1B). ARA70, an AR co-activator, also showed enhancement on ARQ9 and ARQ24. However, the enhancement appeared in the samples in the presence and absence of DHT. There was little or no effect in the samples transfected with ZMIZ2, a homolog of ZMIZ1. We repeated the above transient transfection experiments in PC3, a human prostate cancer cell line, and CV-1 cells, a monkey kidney cell line, and observed the similar results (data not shown). Overall, this was first line of evidence that ZMIZ1, rather than ZMIZ2 or ARA70, enhances the ligand-induced transactivation of AR with shorter polyQ tract in prostate cancer cells.

### ZMIZ1 enhances transactivation of the shorter polyQ AR at physiologic androgen concentration

**Figure 2:**
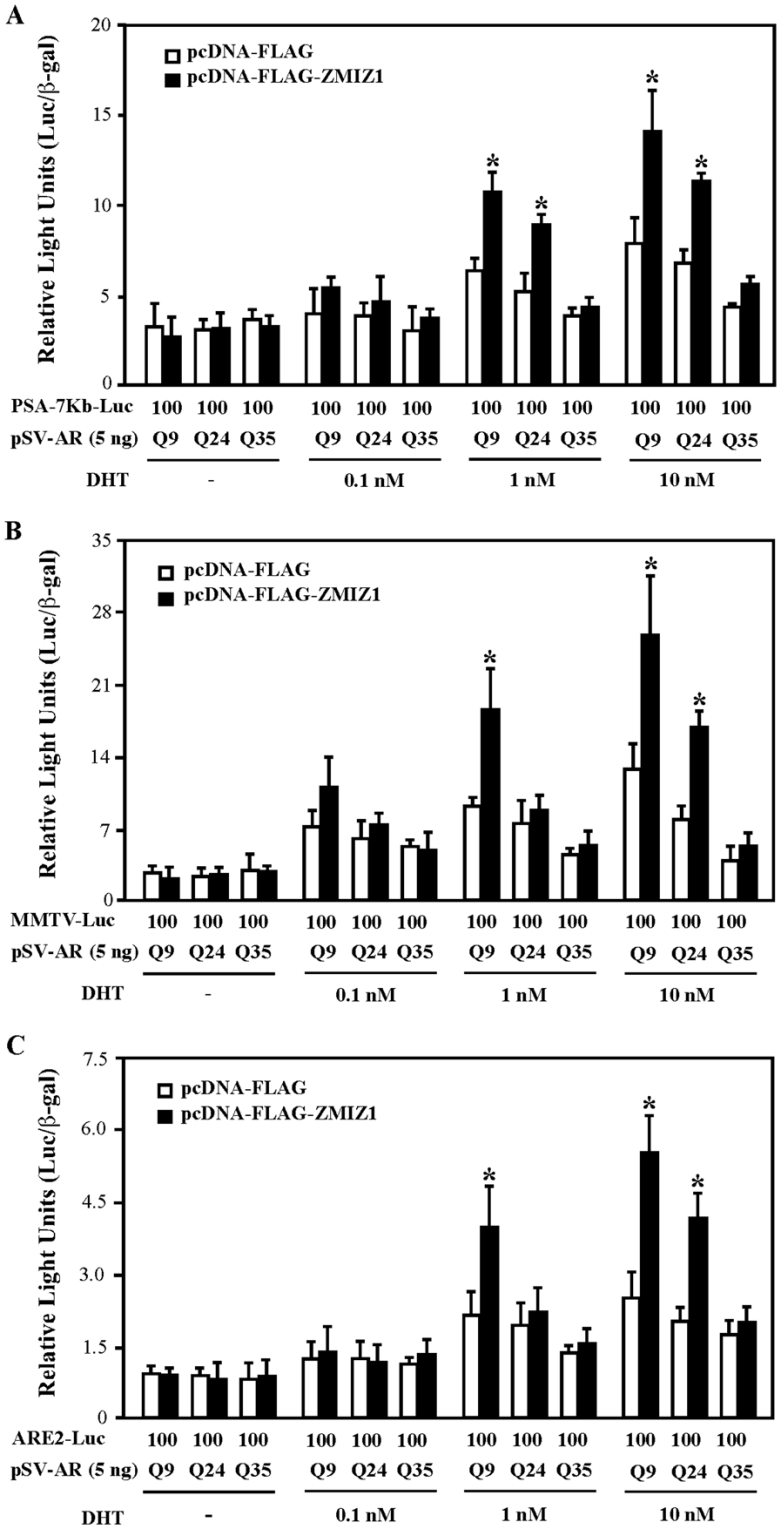
ZMIZ1 enhances androgen-dependent shorter polyQ-AR mediated transcription. (A) DU145 cells were transiently transfected with 100 ng of 7 kb PSA-Luc, 25 ng of pSV40-β gal, 5 ng of ARQ9, Q24, or Q35, and where indicated, with 10 ng of pcDNA3 empty vector or pcDNA3-FLAG-hZMIZ1. Twenty-four hours after transfection, cells were incubated in the absence of DHT or in the presence of 0.1, 1, or 10 nM of DHT for 24 h. Cell lysates were prepared for assessment of luciferase and β-gal activities. The transfection experiments were repeated three times in triplicate. Relative luciferase units (RLUs) were determined from three independent transfections and are presented as the mean ± SEM of the triplicate transfections. “*” indicates a significant difference (p<0.05) between the samples co-expressed with different AR polyQ and ZMIZ1 proteins in the presence of different concentration of DHT, (B) Similar to (A), except that a MMTV-luc reporter (100 ng) was used. (C) Similar to (A), except that an ARE-luc reporter (100 ng) was used.

To explore the biological relevance of ZMIZ1 on the AR with different polyQ tracts, we expanded our investigation by assessing the effect of ZMIZ1 on different AR polyQ proteins at various ligand concentrations and different androgen-induced promoters/reporters in DU145 cells. Three different androgen-inducible reporters were tested in the experiments, including the 7 kb PSA promoter (PSA-luc), a mouse mammary tumor virus promoter reporter (MMTV-Luc), and a minimum promoter with three ARE-luc reporter. ZMIZ1 showed a significant enhancement of both ARQ9 and Q24 transcriptional activities on the PSA-promoter/reporter in the presence of 1 and 10 nM DHT (Fig. 2A). However, in the presence of 0.1 nM DHT, only slight induction was observed in all of the samples transfected with the three different AR polyQ proteins. Expression of ZMIZ1 significantly increased ARQ9 activity on the MMTV- and ARE-promoters/reporters in the presence of 1 and 10 nM DHT but only affected ARQ24 in the presence of 10 nM DHT (black bars, Fig. 2B and 2C). Particularly, the samples co-expressed with ZMIZ1 and ARQ9 in the presence of 10 nM DHT displayed the highest ligand-induced activity on the MMTV-promoter/reporter. The above data demonstrated that ZMIZ1 preferably enhances the activity of AR proteins containing short polyQ lengths on three different androgen induced promoters in the presence of physiologic levels of androgens in prostate cancer cells.

### Enhancement of shorter polyQ AR mediated transcription by ZMIZ1 is associated with the components of SWI/SNF complexes, Brg1 and BAF57

**Figure 3:**
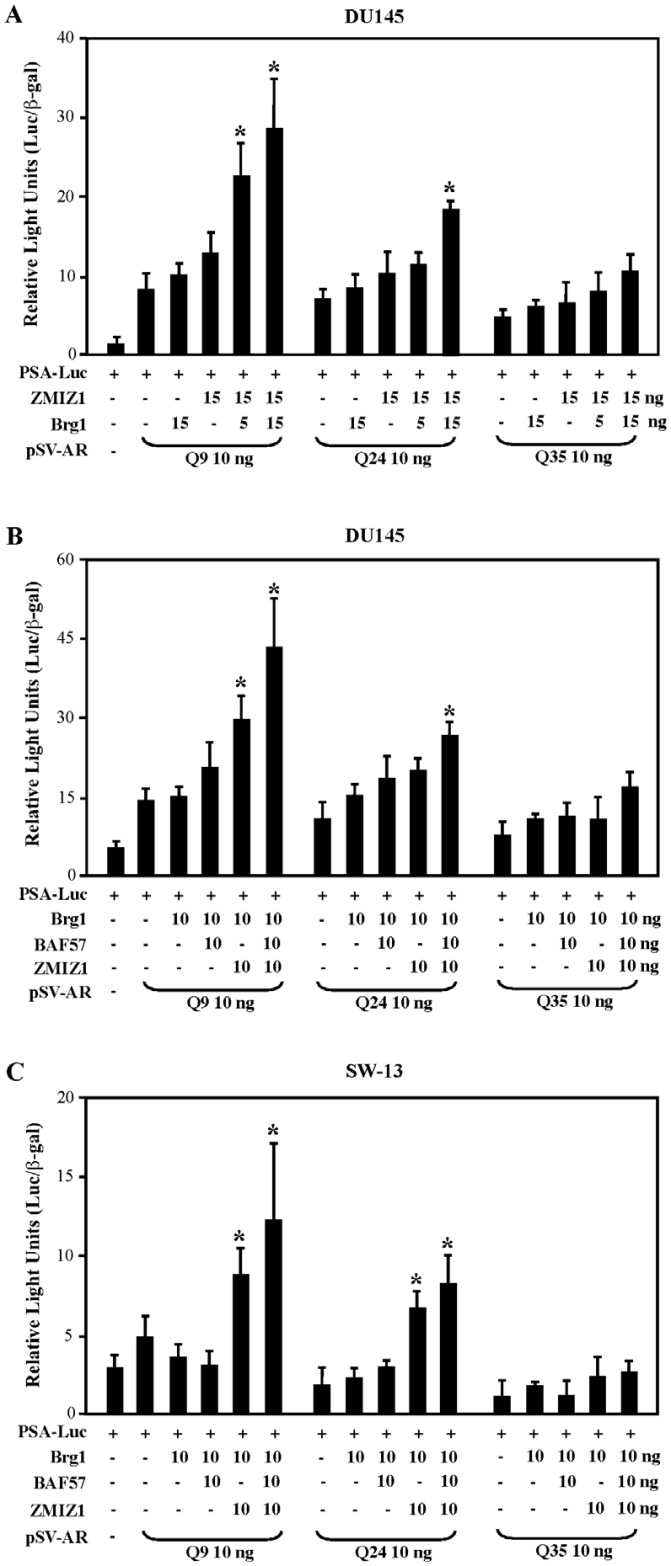
The enhancement of ZMIZ1 on shorter polyQ-AR mediated transcription is associated with SWI/SNF complex, Brg1 and BAF57. (A) DU145 cells were transfected with a luciferase reporter driven by the human PSA promoter (100 ng), pcDNA3-β-gal (25 ng), 5 ng of ARQ9, Q24, or Q35, and where indicated, with 15 ng of pcDNA3 empty vector or pcDNA3-FLAG-ZMIZ1, different amounts of pcDNA3-Brg1 as indicated. Cells were incubated 24 h after transfection in the presence of 10 nM DHT for 24 h. The transfection experiments were repeated three times in triplicate. Relative luciferase units (RLUs) were determined from three independent transfections and are presented as the mean ± SEM of the triplicate transfections. Statistical analyses showed significant differences (p<0.05) between the samples expressed different AR Q proteins only or co-expressed AR and other co-factors in the presence of DHT, “*”. (B) DU145 cells were transfected with a luciferase reporter driven by the human PSA promoter (100 ng), pcDNA3-β-gal (25 ng), 5 ng of ARQ9, Q24, or Q35, and where indicated, with 10 ng of pcDNA3 empty vector or pcDNA3-FLAG-ZMIZ1, or different amounts of pcDNA3-Brg1 or pcDNA3-BAF57 as indicated. Cells were incubated 24 h after transfection in the presence of 10 nM DHT for 24 hr. Cell lysates were then prepared for assessment of luciferase and β-gal activities. Data were analyzed as described in (A). (C) Similar to (A), except in SW-13 cells.

The *Drosophila* ortholog gene of ZMIZs, tonalli (tna), has been shown to genetically interact with the SWI/SNF and Mediator chromatin remodeling complexes [37]. The interactions between ZMIZ proteins and Brg1 and BAF57, the components of SWI/SNF complexes, have been demonstrated [23], [26]. In addition, the SWI/SNF chromatin-remodeling complexes and p160 co-activators have been shown to interact strongly with AR proteins containing a shortened polyQ length in comparison to the wild type AR [19]. Therefore, we next examined the involvement of the SWI/SNF-like BAF complexes in ZMIZ1 regulated AR transcription. A nearly 2, 3, or 5 fold ligand-induced transactivation was observed in the cells transfected with ARQ35, Q24, or Q9 expression vector plasmids on the PSA luciferase reporter, respectively (Fig. 3A). Whereas co-transfection of the Brg1 or ZMIZ1 alone with three different ARQ expression vectors slightly increased the androgen-induced activity on the PSA luciferase reporter, combined expression of both proteins showed a significant enhancement on ARQ9 mediated transcription in a dose dependent manner in comparison to the samples with ARQ24 or Q35 (Fig. 3A). Using similar approaches, we further tested the potential effect of BAF57, a Brg1-associated protein, in ZMIZ1 regulated enhancement of AR polyQ constructs. As shown in Figure 3B, co-expression of ZMIZ1 with Brg1 alone or plus BAF57 significantly enhanced ligand induced activity of ARQ9 or ARQ24 on the PSA-luc reporter. These results demonstrated that Brg1 or BAF57 cooperatively augment ARQ9 mediated transcription with ZMIZ1.

Next, we further assessed the role of the SWI/SNF-mediated complexes in ZMIZ1 regulated AR activity using the human adrenal carcinoma cell line, SW13, which is deficient in both *BRG1* and *Brm* expression and considered to lack functional SWI/SNF complexes [30]. Overexpression of ARQ9 but not Q24 and Q35 conferred about a one fold ligand-induced activity of the PSA reporter (Fig. 3C). Either transfection of Brg1 alone or combined with BAF57 showed no significant changes in AR activity. However, co-expression of ZMIZ1 with Brg1 or with Brg1 and BAF57 significantly enhanced the activity of ARQ9 and ARQ24 on the PSA promoter/reporter. The data further suggested that the Brg1- and BAF57-regulated AR activity is mediated through ZMIZ1.

### The transactivating effect of ZMIZ1 on shorter polyQ AR confers resistance to androgen antagonists

**Figure 4:**
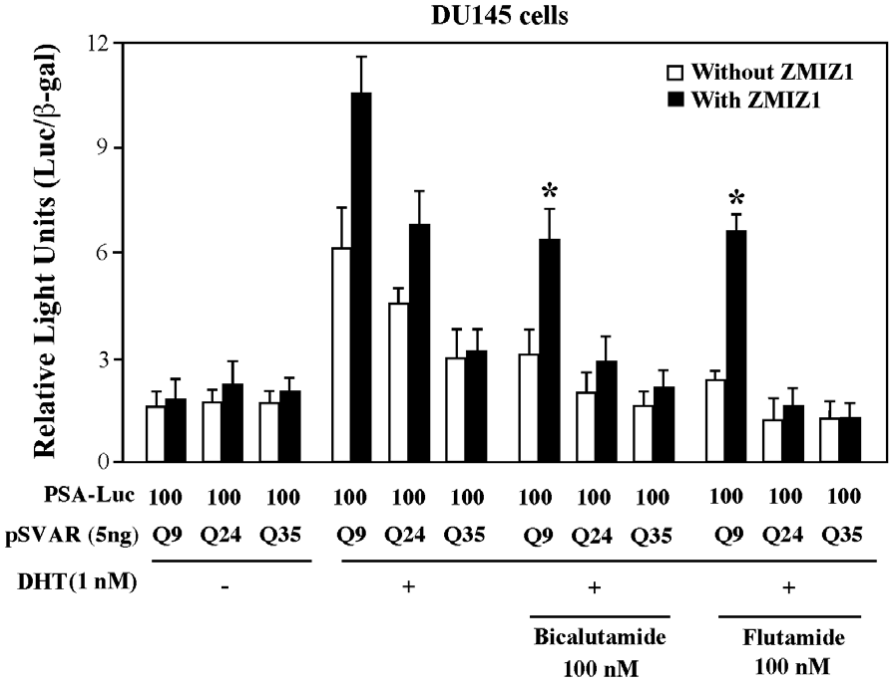
The effect of ZMIZ1 on shorter poly-Q AR is insensitive to androgen antagonist. DU145 cells were transfected with a luciferase reporter driven by the human PSA promoter (100 ng), pcDNA3-β-gal (25 ng), 5 ng of ARQ9, Q24, or Q35, and where indicated, with 10 ng of pcDNA3 empty vector or pcDNA3-FLAG-ZMIZ1. Twenty-four hours after transfection, cells were incubated with different androgen antagonist, including 100 nM of Bicalutamide or Flutamide for 24 h. Cell lysates were prepared for luciferase and β-gal assays. The transfection experiments were repeated three times in triplicate. Relative luciferase units (RLUs) were determined from three independent transfections and are presented as the mean ± SEM of the triplicate transfections. Statistical analyses showed significant differences (p<0.05) between the samples co-expressed with different AR polyQ and ZMIZ1 proteins in the presence of anti-androgens, “*”.

The current literature has shown that the expression of AR and its downstream target genes remain at high levels in castration resistant prostate cancer [14], [38]. A promotional role of AR co-regulators in prostate cancer progression has also been suggested [39], [40]. Here, we examined the effect of ZMIZ1 on different AR polyQ vectors in the presence of antagonists in the prostate cancer cell line, DU145. In the presence of 1 nM DHT, overexpression of ARQ9 and Q24 produced nearly 3 to 2 fold ligand-induced activity on the PSA reporter, respectively (Fig. 4). Approximately 60% and 30% enhancement was generated in the cells co-transfected with ZMIZ1 and ARQ9 or ARQ24 expression vectors. Addition of 100 nM of two anti-androgens, bicalutamide or flutamide, reduced transcriptional activity in DU145 cells transfected with ARQ24 or ARQ35 alone or co-transfected with ZMIZ1. On the contrary, anti-androgens showed less repressive effect on the cells transfected with ARQ9 alone or plus ZMIZ1. Particularly, DU145 cells co-expressed with ARQ9 and ZMIZ1 proteins demonstrated significant resistance to inhibition by antiandrogens in comparison to cells co-expressed ARQ24 and Q35 proteins. We repeated these experiments on PC3 and CV1 cells and observed similar results (data not shown). These data suggest a role for ZMIZ1 mediated enhancement of shorter polyQ AR to escape anti-androgen blockade in prostate cancer cells.

### ZMIZ1 interacts with AR polyQ proteins

**Figure 5:**
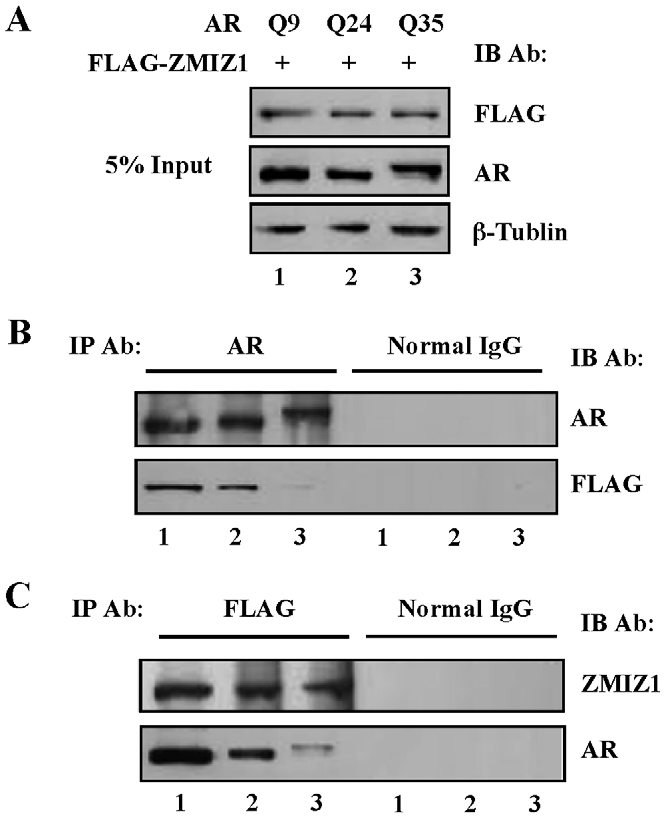
ZMIZ1 preferably binds to the AR protein with a short polyglutamine tract. (A) DU145 cells stably expressing different polyQ AR proteins were transfected with pcDNA3-FLAG-ZMIZ1. Cell lysates were harvested and then immunoprecipitated with anti-FLAG antibody or normal mouse IgG. 5% input of the cell lysates was probed with the anti-FLAG, AR, or Tublin antibody. (B) Immunoprecipitates pulldowned by the AR antibody or normal IgG (IP) were analyzed by Western bolt and probed by AR and FLAG antibodies. (C) Immunoprecipitates pulldowned by the FLAG antibody and IgG (IP) were analyzed by Western blot and probed by ZMIZ1 or AR antibody.

To search for the potential mechanisms by which ZMIZ1 enhances shorter polyQ AR mediated transcription, we carried out co-immunoprecipitation assays to examine the interaction between ZMIZ1 and different AR polyQ proteins. We expressed FLAG-tagged ZMIZ1 together with different AR polyQ proteins in DU145 cells. Whole cell lysates containing almost equal amounts of proteins were immunoprecipitated with normal rabbit IgG or an anti-AR antibody (Fig. 5A). As shown in Fig. 5B, similar levels of ARQ9, Q24, and Q35 were shown in the anti-AR antibody bound immunoprecipitates, which migrated differently in SDS-PAGE gels due to the length of polyQ tracts. Intriguingly, the FLAG-tagged ZMIZ1 protein band appeared more intense in the immunoprecipitate sample with ARQ9 expression than samples with ARQ24 or Q35. To confirm our finding, we performed reverse immunoprecipitation experiments with the FLAG antibody. Whereas nearly equal amounts of FLAG-ZMIZ1 proteins were detected in all of samples with a ZMIZ1 antibody, the ARQ9 showed stronger staining with the AR antibody than ARQ24 or ARQ35 proteins. Taken together, the above data indicate that ZMIZ1 interacts more strongly with the shorter polyQ AR in prostate cancer cells.

### ZMIZ1 affects the interactions between NH2- and carboxyl terminus of AR

**Figure 6:**
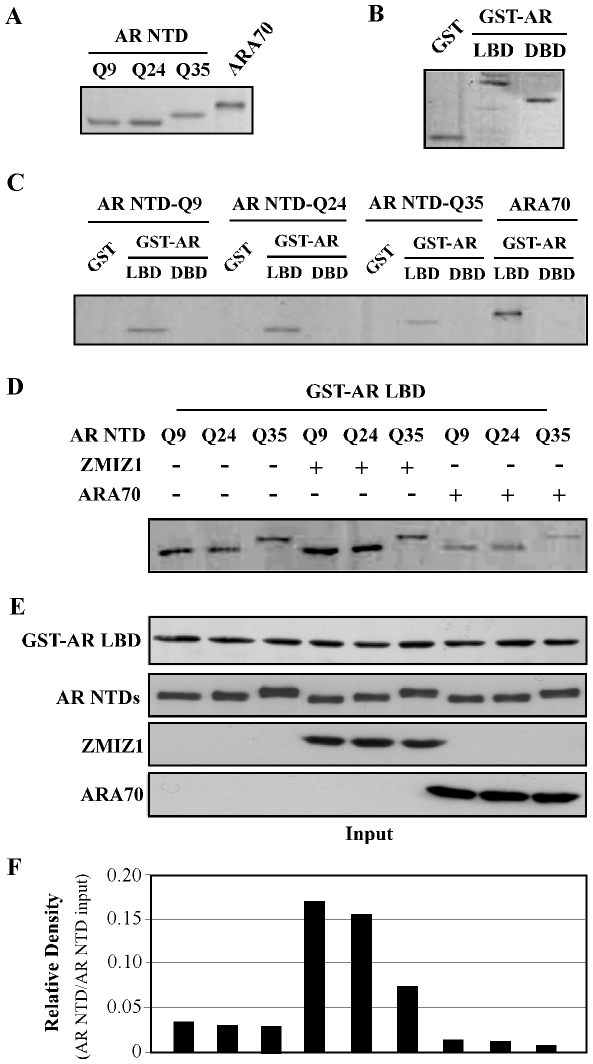
ZMIZ1 enhances the interaction between the N- and C- terminuses of AR. (A) Equal amounts of *in vitro* translated ^35^S methionine-labeled NTD domain of ARQ9, Q24, Q35 or ARA70 were measured and used in GST pull down assays. (B) GST-AR LBD (amino acids: 676–919), GST-AR DBD (aa: 559–624), or GST protein alone, as a negative control, were isolated, purified, and subjected to binding assays. Equal amounts of the above GST proteins were analyzed on SDS-PAGE. (C) Equal amounts of the *in vitro* translated NTD of ARQ9, Q24, Q35 or ARA70 were incubated with different GST fusion proteins coupled to glutathione-Sepharose beads in the binding buffer (see the “Material and Methods) with 10 nM DHT for 4 hours at 4°C. The beads were washed and subjected to SDS-PAGE and autoradiography. (D) DU145 cells with stable expression of different AR polyQ proteins were transfected with pcDNA3-FLAG-ZMIZ1, pcDNA3-FLAG-ZMIZ2FLAG, or pcDNA3 plasmids. Whole cell lysates were prepared from the above cells and subjected to GST pulldown experiments. Forty µl of GST-AR LBD beads were mixed with 200 µl of whole cell lysates isolated from the above DU145 cells and incubated with the binding buffer (see the “Material and Methods) with 10 nM DHT for 4 hours at 4°C. The GST beads were washed, eluted, and subjected to SDS-PAGE and immunoblotting with an anti-AR antibody (sc-7305; Santa Cruz Biotechnology, Santa Cruz, CA). (E) Ten µl of the above cell lysates after mixed with GST-AR LBD were analyzed by Western blotting to assess levels of input from different AR polyQ, FLAG-ZMIZ1 and ARA70 proteins with anti-AR C-terminus (sc-815, Santa Cruz Biotechnology), anti-AR N-terminus (sc-816, Santa Cruz), or anti-FLAG (cat#3165, Sigma) antibody, respectively. (F) Densitometry of the membrane bands was performed, and the relative numbers were reported, as the density of the binding of AR NTD (D) normalized by the input of AR NTD proteins (the second top panel, E).

It has been shown that the intramolecular interaction between the AR NTD and LBD is essential for forming a composite binding site to recruit transcriptional co-regulators to confer the full transcriptional activity [41]. The short polyQ length can enhance the ligand-induced N to C-terminal intramolecular interaction [19]. Therefore, we assessed the potential role of ZMIZ1 on the N/C interaction of AR with different polyQ tracts. We first examined the interactions between the NTDs with different polyQ repeats and the AR DBD or LBD using GST pull-down assays. The GST fusion proteins containing the AR DBD and LBD were generated (Fig. 6B), and incubated with equal amounts of the truncated NTDs with different polyQ tracts generated *in vitro* using the TNT-coupled reticulocyte lysate systems (Fig. 6A). The protein-protein interactions were analyzed by SDS-PAGE and autoradiography (Fig. 6C). Specific retentions of the AR NTDs were observed in the samples incubated with GST-AR LBD protein as well as ARA70, used as a control. Both ARQ9 and ARQ24 showed a strong interaction with the AR LBD in comparison to ARQ35, which is consistent with the previous report [19]. Next, we analyzed the effect of ZMIZ1 on the AR N/C terminal interactions. The GST-AR LBD showed a specific retention with three AR NTD proteins (Fig. 6D). In the presence of ZMIZ1, the interactions between the AR LBD and the NTDs of ARQ9 and ARQ24 were increased in comparison to ARQ35. However, there was only slight change between the samples co-expressed with ARA70 and AR polyQ proteins (Fig. 6E). Measurements of binding by densitometry showed enhancement of N/C interaction in ARQ9 and ARQ24 by ZMIZ1 (Fig. 6F).

**Figure 7:**
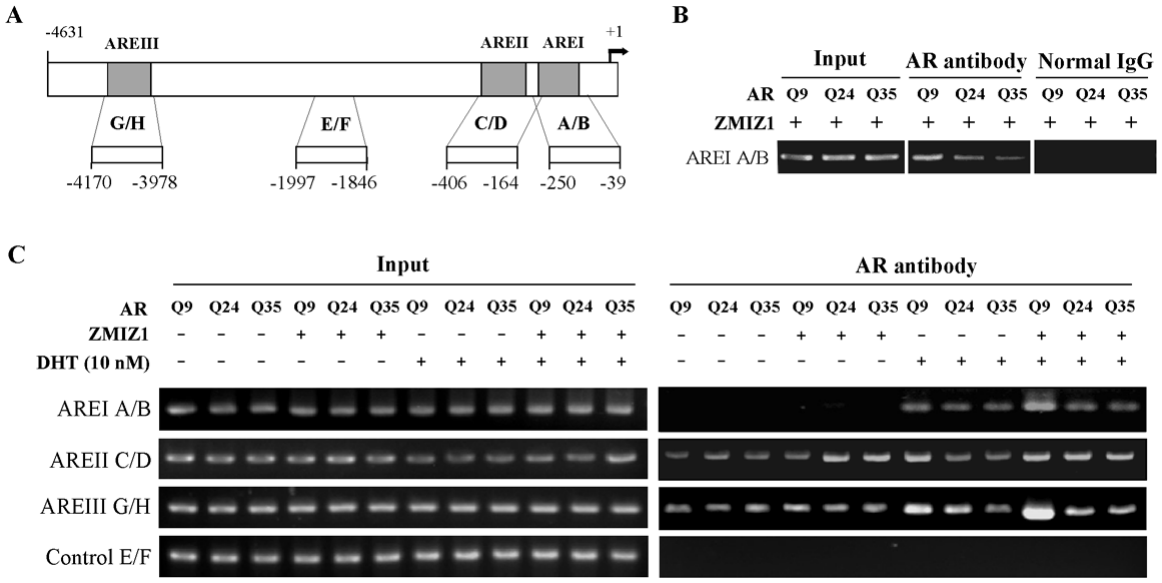
ZMIZ1 enhances shorter polyQ-AR binding to PSA promoter. (A) Schematic diagram of the human PSA promoter region. Four pairs of PCR primers marked as A/B, C/D, E/F, and G/H are used to amplify specific AREs. (B) DU145 cells stably transfected with 7 Kb-PSA-promoter plasmids and expressing different polyQ AR were transfected with pcDNA3-FLAG-ZMIZ1 and incubated in the presence or absence of 10 nM DHT. Soluble chromatin was prepared from formaldehyde-cross-linked and sonicated cell cultures. Specific antibodies against AR (Cat# Sigma) or normal IgG as controls were used to immunoprecipitate protein-bound DNA fragments. These fragments were amplified by PCR using primers A and B. (C) The above soluble chromatin was systemically analyzed by PCR with four pairs of primers as indicated in the figure.

Next, we performed chromatin immunoprecipitation (ChIP) assays to evaluate the effect of ZMIZ1 on AR-occupied promoters. DU145 cells stably transfected with 7 kb-PSA promoter-luciferase reporter plasmids as well as different AR polyQ with or without ZMIZ1 expression vector were used in the experiments. Almost equal levels of exogenous AR and ZMIZ1 proteins were observed by Western blotting in each subline with expression of different AR polyQ protein (Figure S2A). Soluble chromatin was isolated from the formaldehyde treated DU145 cells, sheared by sonication, and immunoprecipitated by anti-AR antibodies or normal IgG as controls. The genomic DNA samples were isolated and then analyzed by PCR using specific pairs of primers spanning the AR-binding sites in the PSA promoter (Figure 7A). We first assessed a specific recruitment of AR on the AREI element using the primer pair labeled as A/B. As shown in Figure 7B, the PCR fragments containing ARE1 were only detected in the immunoprecipitates bound with the AR antibody but not in the controls with normal IgG. PCR fragments showed an increase in the ARQ9 expressed cells in comparison to ones with ARQ24 and ARQ35 (Fig. 7B). Using similar approaches, we then evaluated the effect of ZMIZ1 on the recruitment of different AR polyQ proteins on three ARE sites within the *PSA* promoter region (Fig. 7A). The intensities of PCR fragments containing AREI, AREII, and AREIII were similar or slightly increased in samples co-expressed with ARQ9 in comparison to samples with ARQ24 and ARQ35. However, in the presence of ZMIZ1, a marked increase in the AREI and AREIII PCR fragments was observed in the DHT-induced ARQ9 expressed cells (Fig. 7C). We confirmed these findings by reciprocally immunoprecipitating ZMIZ1 using the same soluble chromatin samples. A significant increase of AREI and AREIII binding was observed in samples co-expressed with ARQ9 and ZMIZ1 in comparison to ARQ24 or ARQ35 and ZMIZ1 (Figure S2B). We also performed qPCR to quantitate the bindings using the AR antibody bound DNA and input DNA samples as templates (Figure S3). A significant increase of ARQ9 binding to AREI and AREIII sites was observed in the samples co-expressing ZMIZ1 in comparison to other samples. These data demonstrated that ZMIZ1 selectively enhances the recruitment of shorter polyQ AR onto the *PSA* promoter, resulting in stronger binding to AREI and AREIII sites.

## Discussion

The importance of the androgen receptor has been implicated in both normal prostate development and prostate cancer pathogenesis. The AR mediates most of its action through regulation of transcription of its downstream target genes. Unlike other nuclear hormone receptors, the AR contains a relatively weak activation function 2 (AF2) domain within the ligand-binding domain (LBD). The NTD is coded by exon 1 and does not share homologies with other steroid receptor family members [2]. The NTD contains two transactivation regions. The main transactivation region (AF-1) in the NTD spans between amino acids 51 and 211, which encompasses the polyQ tract. Very similar polyQ tracts have been identified in other transcription factors such as the TATA box binding protein (TBP) [42]. Previous studies have suggested that the polymorphic polyQ tract of the AR NTD might be involved in the control of AR mediated transcription by facilitating protein–protein interactions with other transcriptional factors [17], [19], [20]. Previously, we demonstrated that ZMIZ1 is an AR interacting protein. ZMIZ1 binds primarily to the region between amino acids 243–333, which is between two AF regions within the NTD [21]. In this study, we showed that ZMIZ1 binds to the AR polyQ9 proteins much stronger than AR polyQ24 and polyQ35 in the immunoprecipitation assays. We also observed a pronounced effect of ZMIZ1 in enhancing the N- and C- terminal interaction of AR with short polyQ tracts. It has been suggested that the intramolecular interaction between the AR NTD and LBD is essential for forming a composite binding site to recruit transcriptional co-regulators to confer the full transcriptional activity [19], [41]. Therefore, our findings suggest that ZMIZ1 facilitates a strong interaction between the N- and C-terminus of short polyQ AR that leads to recruitment of other transcriptional co-regulators and activate transcription.

Previously, we demonstrated that both ZMIZ1 and ZMIZ2 interact with AR and enhance AR mediated transcription [23]. These two proteins share significant sequence similarity, particularly within their C-terminal regions [26]. They contain an intrinsic transactivation domain and function as transcriptional co-activators [21], [26]. These two ZMIZ proteins show different tissue distribution profiles, suggesting distinct roles for these proteins in regulating the expression of different target genes. In this study, we observed a remarkable enhancement of ARQ9 mediated transcription by ZMIZ1 and not ZMIZ2. These observations further suggested that ZMIZ1 and ZMIZ2 proteins, although structurally similar, likely play different roles in modulating transcription. In addition, ARA70, another AR associated protein, did not show a significant effect on ligand-induced transcription of different polyQ length AR proteins. These observations further implicate that the enhancement by ZMIZ1 on short polyQ AR proteins is a specific effect and needs to be further evaluated.

A homologue of ZMIZ1 and ZMIZ2, termed *tonalli* (*tna*) was identified previously in *Drosophila* [37]. The protein encoded by *tna* was shown to interact with SWI2/SNF2 and the Mediator complex, suggesting a potential role for the ZMIZ proteins in chromatin modification. Previously, we demonstrated that the ZMIZ proteins interact with both Brg1 and BAF57, DNA-binding subunits of the mammalian SWI/SNF-like BAF complexes that enhance AR mediated transcription [43]. It has been shown that BAF57 can directly bind to the AR and be recruited to the endogenous AR target promoter upon ligand activation [44], [45]. Therefore, we assessed the involvement of Brg1 and BAF57 in ZMIZ1 mediated enhancement of AR activation. Using SW13 cells, which were deficient in both *BRG1* and *Brm* expression and considered to lack the functional SWI/SNF complexes [30], we demonstrated the requirement of both Brg1 and BAF57 in the enhancement of ZMIZ1 on ARQ9 mediated transcription. The observation provides additional evidence that the mammalian SWI/SNF-like BAF complexes play a role in ZMIZ1-mediated AR activation.

Different mutations in the *AR* gene have been identified in various human disorders, including endocrine dysfunctions and prostate cancer. Most loss-of-function mutations in the *AR* gene result in the inactivation of AR function through impairing the androgen-dependent transcription [8]. Particularly, extended lengths of polyQ repeats within the AR NTD can result in dysfunctional AR and have been identified in patients with androgen insensitivity syndrome, (AIS). In contrast, gain-of-function mutations have been identified in prostate cancers and hirsutism in females [6], [7], [46]. While the molecular mechanisms underlying these mutations in the pathogenesis of the above human disorders are unclear, the inverse correlation between the length of polyQ tracts and the transcriptional activity of AR has been observed in prostate cancer patients [3], [5], [12]. In this study, we confirmed the previous observations that the AR containing the shorter length of polyQ tracts has the higher transcriptional activity on three different androgen-inducible promoter/reporter constructs. Most importantly, we observed much stronger enhancement by ZMIZ1 on ARQ9 mediated transcription in comparison to ARQ24 or ARQ35. ZMIZ1 contains a very strong intrinsic transcriptional domain in the C-terminal proline rich domain. Through specific protein-protein interaction, ZMIZ1 can be recruited into the AR-involved transcriptionally active complexes to facilitate AR mediated transcription. We used chromatin immunoprecipitation assays (ChIP) to demonstrate a specific recruitment of ZMIZ1 on the androgen response elements with the *PSA* promoter region. Since the full transcriptional activity of AR is conferred through other co-activators, the above data suggest a potential mechanism underlying the short length of polyQ tracts in enhancing ligand-induced AR-mediated transcription. Therefore, it would be interesting to characterize the expression of ZMIZ1 and different length polyQ AR in patient samples of prostate cancer, AIS, and other human disorders that may be closely related to either loss or gain-of-function mutations in the *AR* gene.

In this study, we demonstrated on all three androgen-induced promoters that ZMIZ1 showed maximal induction on ARQ9 mediated transcription at either 1 or 10 nM of DHT, most close to the physiological level in humans. These observations suggest the enhancement by ZMIZ1 on the short polyQ AR activity represents a biologically relevant event. The humanized AR mouse lines with different lengths of polyQ tracts have been generated using germline gene targeting [47]. An inverse correlation of polyQ tract length with AR transcriptional activity on its target genes, such as probasin and Nkx3.1, was observed in the mouse prostate tissues. It would be interesting to investigate the role of ZMIZ1 on AR with different polyQ tracts using the relevant mouse models. Intriguingly, our transient transfection experiments showed that the enhancement by ZMIZ1 on ARQ9 was not inhibited by anti-androgens at 100 nM. Previous studies have shown that these antiandrogens effectively decrease AR-mediated transcription at this concentration using the similar transient transfection assays [48]. Our findings raise an interesting question regarding the potential significance of ZMIZ1 effects on AR mediated transcription in prostate cancer pathogenesis and particularly in disease progression during androgen deprivation therapy. Further studies into the molecular mechanisms by which ZMIZ1 regulates AR mediated transcription may provide new insight into the biological role of ZMIZ1 in prostate cancer and related human disorders.

## Supporting Information

Figure S1 **Whole cell lysates were isolated from human prostate cancer cells, DU145, which were transiently transfected with 100 ng of 7 kb PSA-Luc, 25 ng of pSV40-β-gal, 5 ng of ARQ9, Q24, or Q35, and 10 or 30 ng of pcDNA3-FLAG-hZMIZ1, pcDNA3-FLAG-hZMIZ2, or pcDNA3-FLAG-ARA70 in the presence 10 nM DHT (see** **Figure 1** **).** Western blotting was performed to assess the expression of AR and other co-activators. Twenty µl of whole cell lysates were eluted on a 10% SDS-PAGE, and transferred onto a nitrocellulose membrane. Membranes were probed with antibodies against AR, FLAG, or β-tublin at the appropriate dilutions. Anti-rabbit or mouse IgG conjugated to horseradish peroxidase was used as secondary antibodies (Bio-Red). Detection was performed with ECL reagents according to the manufacturer's protocol using ECL Hyperfilm (Amersham Biosciences).
(TIF)

Figure S2 **DU145 cells stably transfected with 7 Kb-PSA-promoter plasmids and expressed different AR polyQ proteins were transfected with pcDNA3-FLAG-ZMIZ1 for ChIP analyses.** Whole cell lysates were prepared and 10 ml was used to assess levels of AR and ZMIZ1 proteins by Western blotting. Almost equal amounts of AR and ZMIZ1 as well as β-tublin, used as a control, were detected in the different cell samples (A). Soluble chromatin was isolated from the above cells and subjected to ChIP assays. FLAG antibodies (Cat#F3165 Sigma) were used to immunoprecipitate FLAG-ZMIZ1-bound DNA fragments. Four pairs of primers (see Figure 7A) were used to amplify the regions containing the ARE sites (B).
(TIF)

Figure S3 **DNA templates isolated by immunoprecipitating chromatin by the AR antibody (see the Materials and Methods and** **Figure 7** **) and input DNA samples were analyzed using Q-PCR with specific primers.** The location of primers within the PSA promoter are indicated in Figure 7A. Amplification was performed using 12.5 µl of SYBR qPCR Super Mix Universal (Invitrogen), 10 µM of each primer and 100 ng of DNA in a final volume of 25 ml in the MX 3005P thermocycler (Stratagene). Amplification conditions included an initial denaturation at 95°C for 10 min, followed by 30 cycles at 95°C for 15 s, 55°C for 30 s, and 72°C for 30 s. Serial dilutions of control DNA ranging from 200 to 0.02 ng were used for quantification of the signal. The levels of amplification with different primers for AREI (A), AREII (B), and AREIII (C) were expressed as the fold change of the input chromatin fraction defined by the following equation: immunoprecipitated chromatin fraction with AR antibody/input chromatin fraction. The PCR reactions were repeated three times in triplicate. The fold changes were determined from three independent transfections and are presented as the mean ± SEM. “*” indicates significant differences (p<0.05) between samples co-expressing different AR Q proteins and ZMIZ1.
(TIF)
