# Supplementary figures and images for: ZMIZ1 Preferably Enhances the Transcriptional Activity of Androgen Receptor with Short Polyglutamine Tract
Source: PLoS One. 2011 Sep 20;6(9):e25040. doi: 10.1371/journal.pone.0025040 (PMC3176788; doi:10.1371/journal.pone.0025040)

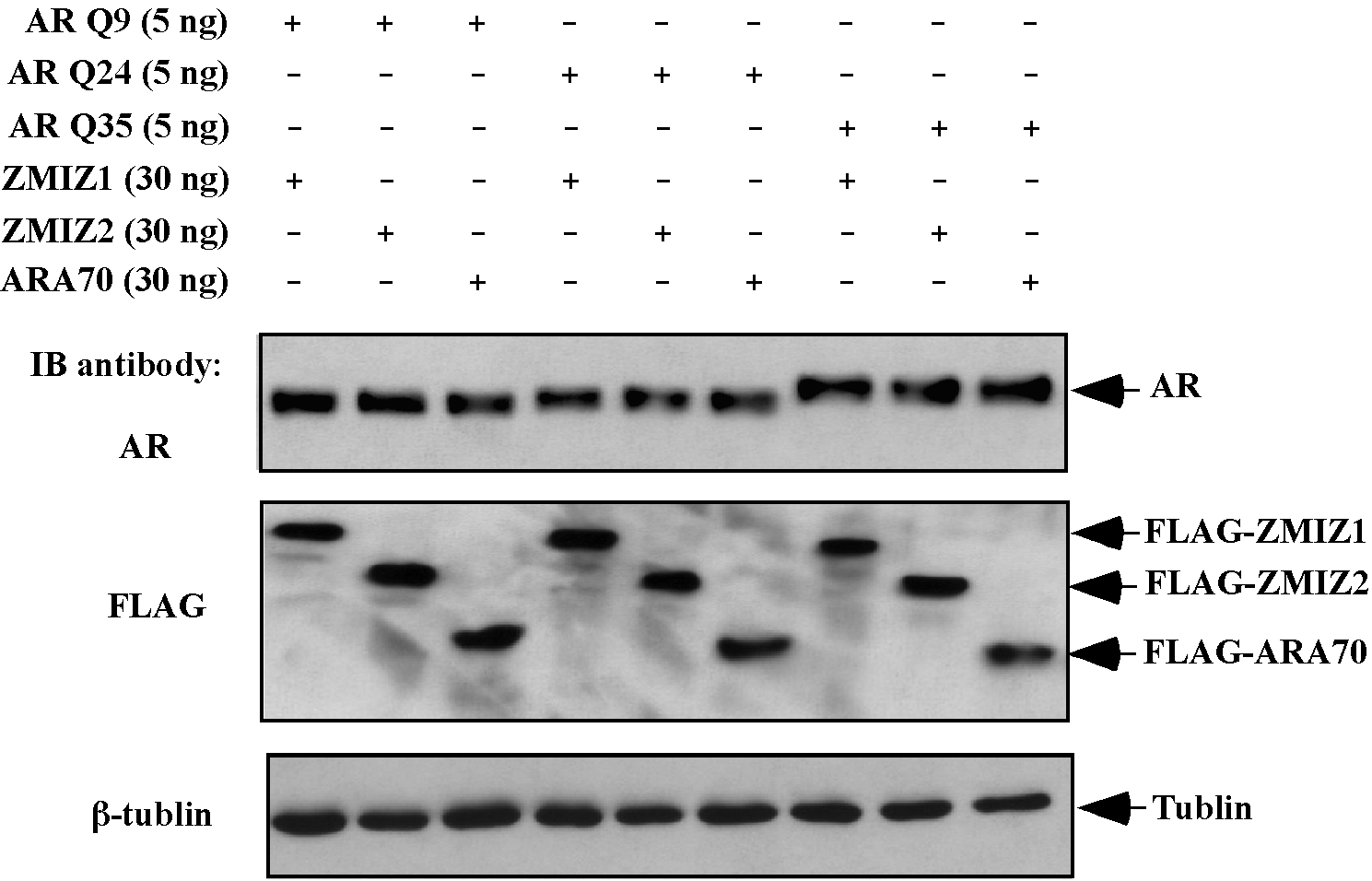

Supplement: Figure S1 — Whole cell lysates were isolated from human prostate cancer cells, DU145, which were transiently transfected with 100 ng of 7 kb PSA-Luc, 25 ng of pSV40-β-gal, 5 ng of ARQ9, Q24, or Q35, and 10 or 30 ng of pcDNA3-FLAG-hZMIZ1, pcDNA3-FLAG-hZMIZ2, or pcDNA3-FLAG-ARA70 in the presence 10 nM DHT (see Figure 1 ). Western blotting was performed to assess the expression of AR and other co-activators. Twenty µl of whole cell lysates were eluted on a 10% SDS-PAGE, and transferred onto a nitrocellulose membrane. Membranes were probed with antibodies against AR, FLAG, or β-tublin at the appropriate dilutions. Anti-rabbit or mouse IgG conjugated to horseradish peroxidase was used as secondary antibodies (Bio-Red). Detection was performed with ECL reagents according to the manufacturer's protocol using ECL Hyperfilm (Amersham Biosciences). (TIF) [file pone.0025040.s001.tif]

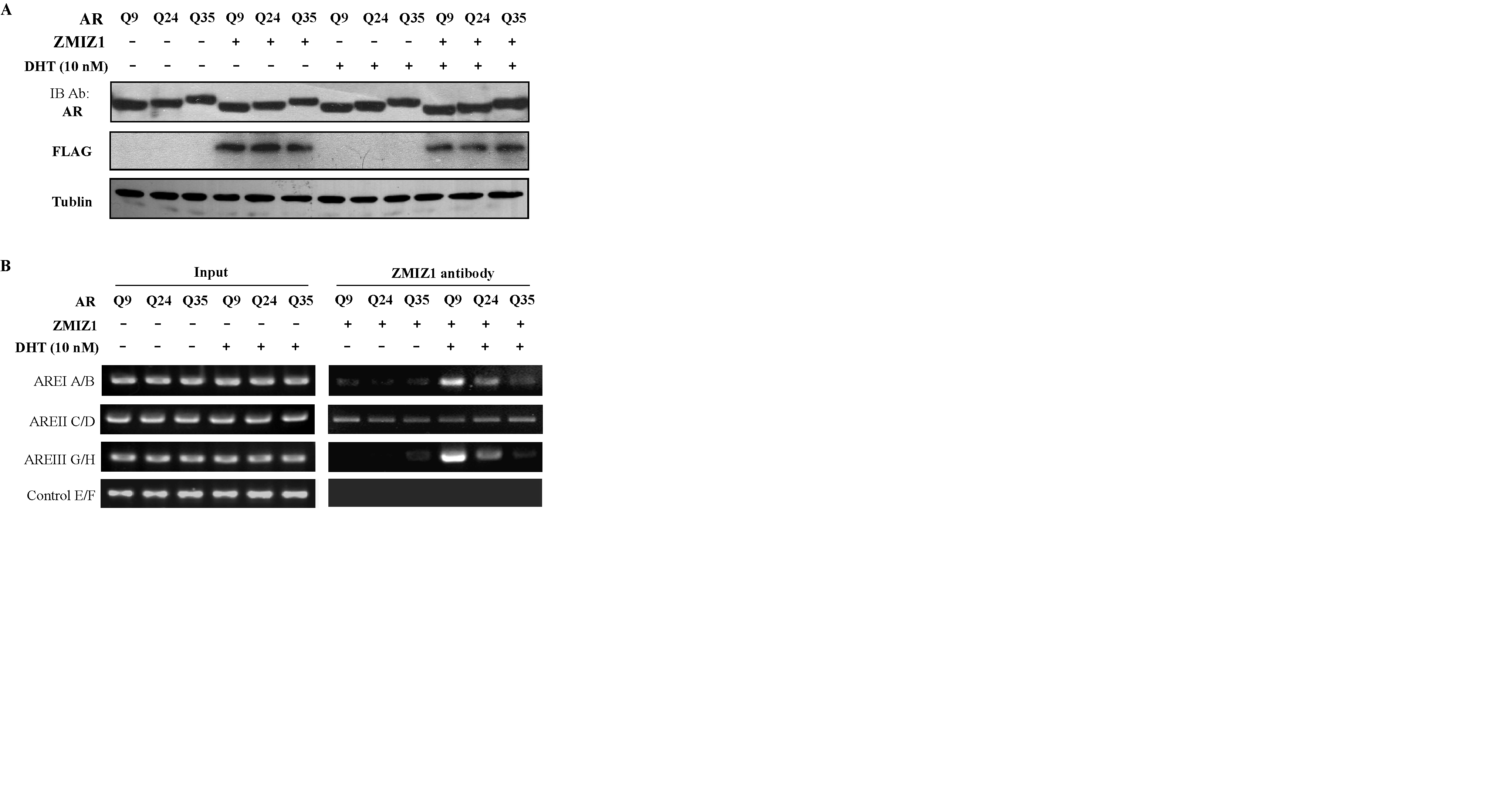

Supplement: Figure S2 — DU145 cells stably transfected with 7 Kb-PSA-promoter plasmids and expressed different AR polyQ proteins were transfected with pcDNA3-FLAG-ZMIZ1 for ChIP analyses. Whole cell lysates were prepared and 10 ml was used to assess levels of AR and ZMIZ1 proteins by Western blotting. Almost equal amounts of AR and ZMIZ1 as well as β-tublin, used as a control, were detected in the different cell samples (A). Soluble chromatin was isolated from the above cells and subjected to ChIP assays. FLAG antibodies (Cat#F3165 Sigma) were used to immunoprecipitate FLAG-ZMIZ1-bound DNA fragments. Four pairs of primers (see Figure 7A) were used to amplify the regions containing the ARE sites (B). (TIF) [file pone.0025040.s002.tif]

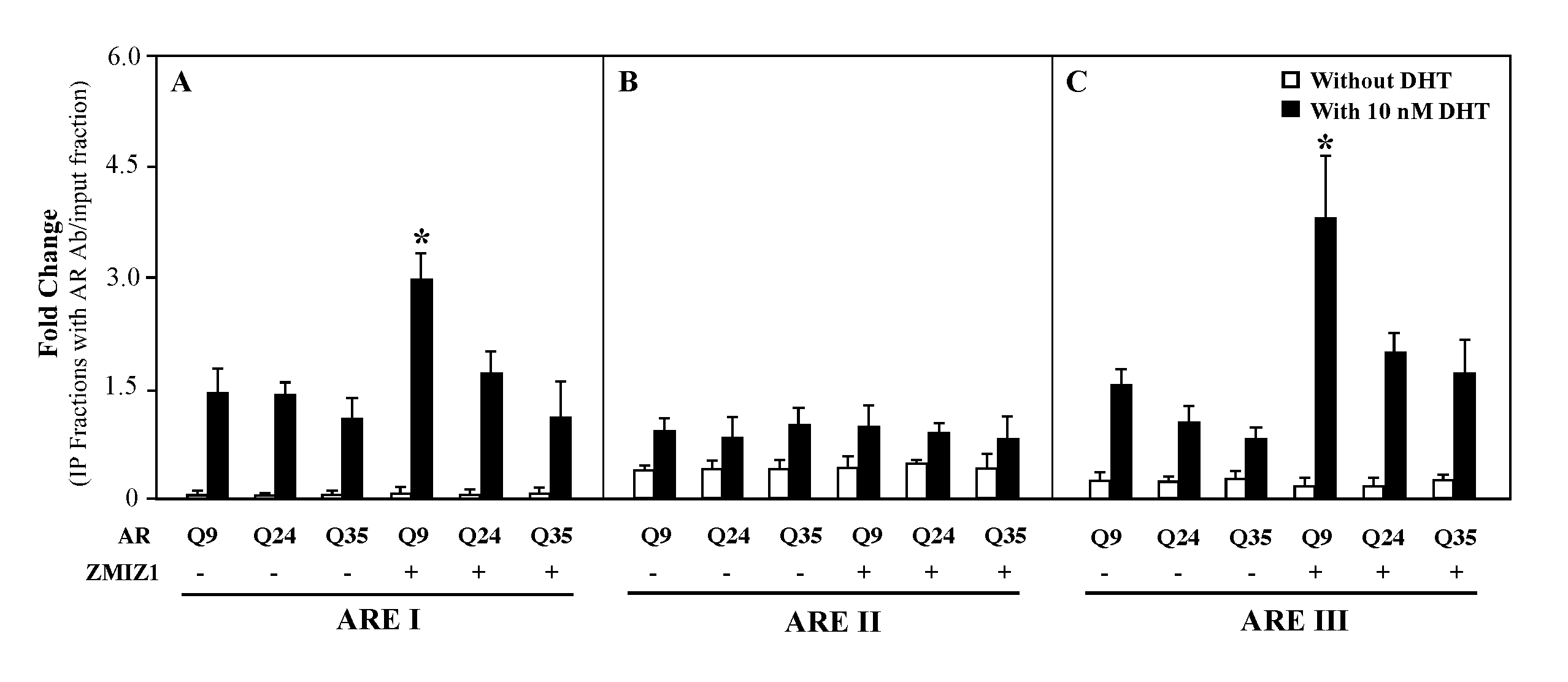

Supplement: Figure S3 — DNA templates isolated by immunoprecipitating chromatin by the AR antibody (see the Materials and Methods and Figure 7 ) and input DNA samples were analyzed using Q-PCR with specific primers. The location of primers within the PSA promoter are indicated in Figure 7A. Amplification was performed using 12.5 µl of SYBR qPCR Super Mix Universal (Invitrogen), 10 µM of each primer and 100 ng of DNA in a final volume of 25 ml in the MX 3005P thermocycler (Stratagene). Amplification conditions included an initial denaturation at 95°C for 10 min, followed by 30 cycles at 95°C for 15 s, 55°C for 30 s, and 72°C for 30 s. Serial dilutions of control DNA ranging from 200 to 0.02 ng were used for quantification of the signal. The levels of amplification with different primers for AREI (A), AREII (B), and AREIII (C) were expressed as the fold change of the input chromatin fraction defined by the following equation: immunoprecipitated chromatin fraction with AR antibody/input chromatin fraction. The PCR reactions were repeated three times in triplicate. The fold changes were determined from three independent transfections and are presented as the mean ± SEM. “*” indicates significant differences (p<0.05) between samples co-expressing different AR Q proteins and ZMIZ1. (TIF) [file pone.0025040.s003.tif]
